# Nutritional influences on human neurocognitive functioning

**DOI:** 10.3389/fnhum.2014.00358

**Published:** 2014-05-27

**Authors:** Michael A. Smith, Andrew B. Scholey

**Affiliations:** ^1^Department of Psychology, Faculty of Health and Life Sciences, Northumbria UniversityNewcastle upon Tyne, UK; ^2^Centre for Human Psychopharmacology, Swinburne University of TechnologyMelbourne, VIC, Australia

**Keywords:** nutrition, diet, brain, neurocognitive functioning, neuroimaging

The notion that good nutrition is essential for adequate growth and sound physical wellbeing is very well established. Further, in recent years, there has been an overwhelming increase in research dedicated to better understanding how nutritional factors influence cognition and behavior (Riby et al., 2012). An aim of this Research Topic was to bring together Review, Opinion and Original Research articles reflecting the current science in this discipline. These include the effects of a range of foods and nutritional substrates on acute and chronic human neurocognitive functioning. The 13 accepted papers which form this Research Topic cover a diverse range of topics relating nutritional factors to neurocognitive functioning and performance. The articles demonstrate that neurocognitive performance is influenced by nutritional factors ranging from the dietary level (e.g., whole diet and meal composition) through to effects of macronutrients (such as glucose and omega-3 fatty acids) and micronutrients (vitamins, iron) on neurocognitive performance.

An objective of this research topic was to consider how various nutritional factors impact upon neurocognitive functioning at different stages of the lifespan. A number of the submissions focused on effects of nutrition in childhood, during which time nutrition plays an important role in growth and development, including via influences on constituents of the human central nervous system. A review by Nyaradi et al. (2013) considered the role of nutrition from a very broad perspective on neurocognitive development from the prenatal period through to childhood. This suggested that while observational studies have supported an important role for several individual nutrients (such as omega-3 fatty acids, B vitamins, iron) in the neurocognitive development of children, intervention studies aimed at supplementing intake of these individual nutrients have demonstrated inconclusive benefits. The authors of this review also highlighted the beneficial neurocognitive effects of breastfeeding and regular breakfast consumption as well as the impairing neurocognitive effects of childhood malnutrition. Kitsao-Wekulo et al. (2013) aimed to extend current understanding of this link between childhood malnutrition and poor cognitive outcomes, by investigating nutritional status as a mediator of the relationship between several socio-demographic variables and cognitive function in a sample of predominantly rural-dwelling Kenyan children. Nutritional status was found to mediate the relationship between socio-demographic factors and (i) language, (ii) motor function, and (iii) executive functioning in this study. With respect to specific micronutrient deficiencies that translate to adverse neurocognitive outcomes, Radlowski and Johnson (2013) reviewed the literature relating to the most common global nutrient deficiency, namely iron deficiency. They report that maternal anemia during the perinatal period increases the risk of delayed neurocognitive development. A further nutrient for which intake is typically below recommended levels in Western individuals is the omega-3 docosahexaenoic acid (DHA). Low dietary levels of this essential fatty acid are potentially problematic given (i) the involvement of this nutrient in mediating several critical brain functions, and (ii) DHA is derived from the diet alone. Similarly to the review of Nyaradi et al. (2013), Heaton et al. (2013) review concludes that dietary and plasma DHA levels in infancy appear to be associated with enhanced cognitive development, but that RCTs investigating infant DHA supplementation have been inconclusive with respect to beneficial effects on cognitive development. However, these authors note substantial methodological issues with RCTs of infant DHA supplementation studies, which could in part explain the equivocal findings (see also Meldrum et al., 2011). In a further review by Whiteley et al. (2013), it was argued that several dietary interventions have been effective in attenuating the neurocognitive and other adverse psychological outcomes in developmental disorders. The authors focused specifically on an intervention involving dietary elimination of gluten (the major protein in wheat, barley and rye) and casein (found in mammalian dairy products), and reported that this gluten and casein free dietary intervention was effective in enhancing such functions as language, attention and motor control in individuals with autism spectrum disorders.

Caroline Edmonds has conducted several studies investigating the influence of hydration status on cognitive functioning, with previous studies observing that access to water improves cognitive performance in children (Edmonds and Jeffes, 2009). In the paper included in this Research Topic, Edmonds et al. (2013) observed that beneficial effects of water consumption may be limited to individuals with relatively higher levels of subjective thirst, with thirsty individuals who were not provided with water exhibiting slower simple reaction times compared with (i) those who were administered water and (ii) those who were not administered water but reported lower levels of subjective thirst. In a further empirical study, Gibson et al. (2013) found that younger women with a higher dietary intake of saturated fat showed deficits in learning and memory.

Three papers accepted into our Research Topic considered the role of breakfast, which has been argued by many nutritionists to be the “most important meal of the day,” in neurocognitive performance. A review by Adolphus et al. (2013) reported that (i) the quality and frequency of the habitual breakfast meal and (ii) engagement with school breakfast programmes in children and adolescents influences academic attainment. In addition Defeyter and Russo (2013) investigated the acute effect of breakfast consumption (compared to fasting) in adolescent non-habitual breakfast consumers, and observed that breakfast consumption enhanced verbal memory (under conditions of greater cognitive load) and backwards counting performance. However, no effects were observed in a range of other cognitive domains. Conversely, Zilberter and Zilberter (2013) highlight the equivocal findings of previous studies investigating the relationship between breakfast consumption and neurocognitive performance. These authors report that several different breakfast effects which have been investigated previously (e.g., glycemic load of the breakfast meal, nutritional composition, breakfast vs. no breakfast) have yielded positive, negative, and null effects on neurocognitive performance across a range of different populations under investigation. Thus it appears that more studies are needed to ascertain the specific benefits of breakfast on neurocognitive performance.

Finally, in recent years neuroimaging studies have made a substantial contribution to our understanding of the neurocognitive mechanisms underpinning nutritional influences on human cognitive performance. Three papers within this Research Topic specifically discuss the role of neuroimaging in investigating the link between nutrition and cognitive functioning. With respect to carbohydrate intake and neurocognitive performance, it is well established that glucose ingestion enhances memory performance, but no such beneficial memory effect of glucose is typically observed for emotionally laden stimuli (Smith et al., 2011). Schopf et al. (2013) report that following glucose ingestion, the hypothalamus becomes inactive in response to emotional material, providing a mechanistic explanation for the previously observed behavioral observations. Further, Jackson and Kennedy (2013) discuss the ways in which near-infrared spectroscopy has proven useful in detecting changes in cerebral blood flow following ingestion of dietary constituents including caffeine, polyphenols and omega-3 fatty acids. A paper which reviewed the literature relating to neuroimaging studies that have investigated the mechanisms underpinning the influence of early diet on cognitive and brain development by Isaacs (2013) provides a sound overview of the work which has been conducted on this topic.

In summary, it is clear that nutritional status, diet and the ingestion of a range of nutrients impacts upon neurocognitive development, function, and performance. The papers within this Research Topic consider a range of these effects. However, equivocal findings have emerged from many studies which have investigated the relationship between nutrition and cognition. Neuroimaging studies are informative with respect to the precise mechanisms which mediate these effects, and future studies in this area will contribute greatly to our understanding of the relationship between nutrition, diet and human neurocognitive functioning.

## Conflict of interest statement

Andrew Scholey has received research funding and consultancy from the health supplement industry. The authors declare that the manuscript was prepared in the absence of any commercial or financial relationships that could be construed as a potential conflict of interest.
